# REDUCING SERIOUS FALL INJURIES IN OLDER ADULTS: RESULTS OF A MULTICENTER WEARABLE TECHNOLOGY STUDY

**DOI:** 10.1093/geroni/igae098.2233

**Published:** 2024-12-31

**Authors:** Bruce Kinosian, Richard Stefanacci

**Affiliations:** Philadelphia Veterans Affairs Medical Center, Philadelphia, Pennsylvania, United States; Thomas Jefferson University, Philadelphia, Pennsylvania, United States

## Abstract

Falls remain an immense public health concern for our growing aging population, causing over 3 million ER visits annually. With falls that result in hip fractures having devastating consequences for older adult health and independence, new solutions are urgently needed. This study evaluated the real-world effectiveness of the Tango® Belt, an innovative wearable device aimed at preventing serious fall injuries. In this six-month, comparative, adaptive study with historical controls conducted across 10 communities located in PA and CA, older adults used sensor-embedded belts alongside standard fall mitigation protocols. When worn during a potentially harmful fall, the belt deploys an airbag to cushion impact. We compared fall outcomes in the belt users (N=134) to propensity score matched historical controls (N=264). Analysis of fall events between the matched groups demonstrated similar rates of falls (p=0.081), but a significant reduction in the proportion of major hip injuries in the intervention versus the control group (1.1% vs 12.1% p=0.004) demonstrating a protective effect of 90.9% against hip fractures/dislocations due to serious hip-impacting falls. Further analysis reveals that the proportion of hospitalizations (8.2% vs 18.9% p=0.004) and ED visits (6.0% vs 17.8% p=0.0001) were also significantly reduced in the intervention vs control group. With compliance recorded at 67% across subjects and no serious AE’s noted related to the device, such “smart garments” address known compliance barriers with passive hip protectors. With serious fall injuries expected to exponentially rise with population aging, the study provides promising evidence for future use of disruptive innovations like Tango Belt.

